# Insulin resistance and obesity: how do they affect tau pathology?

**DOI:** 10.1186/1750-1326-8-S1-P67

**Published:** 2013-10-04

**Authors:** Maud Gratuze, Noura El Khoury, Maya DicklerI, Marie-Amélie Papon, Carl Julien, François Marcouiller, Françoise Morin, Emmanuel Planel

**Affiliations:** 1 Université Laval, Québec, QC, Canada

## 

Tau is a microtubule-associated protein that is abundant in the central nervous system and expressed mainly in axons. Tau hyperphosphorylation can induce its aggregation, and is thought to induce neurofibrillary tangles formation in Alzheimer's disease (AD) and other tauopathies. Understanding the causes and consequences of tau pathology is important because it shows a strong relationship to dementia in AD, and to memory loss in normal aging and mild cognitive impairment.

The causes of sporadic AD are likely to be multifactorial, with external and biological factors interacting with genetic susceptibilities to accelerate the manifestation of the disease. Diabetes and obesity might be such accelerating factors since both are independently linked to cognitive decline, and since diabetes has been shown to increase the risk of AD. It has been suggested that the effects of diabetes and obesity on AD pathogenesis might be mediated by the concommitant insulin resistance that can be present in both medical conditions. However, recent data data suggest that obesity could accelerate tau pathology in the absence of insulin resistance.

To address this controversy, we used genetic models of diabetes and obesity (db/db mice and ob/ob mice) that present different degrees of insulin resistance. We found that both db/db and ob/ob mice had tau hyperphosphorylation, but also mild hypothermia, which is a powerful promoter of tau hyperphosphorylation. Maintaining the mice normothermic resulted in total rescue of tau phosphorylation in db/db mice, but not in ob/ob mice.

Our results suggest that insulin resistance induces tau hyperphosphorylation through hypothermia in both mouse models, but that ob/ob mice have additional upregulation of tau phosphorylation independant of temperature and insulin resistance.

This research will help understanding the link between diabetes, obesity and AD, and the development of future treatments or life style strategies destined to check the advance of the disease.

